# Material recognition based on thermal cues: Mechanisms and applications

**DOI:** 10.1080/23328940.2017.1372042

**Published:** 2017-11-10

**Authors:** Hsin-Ni Ho

**Affiliations:** NTT Communication Science Laboratoires, Nippon Telegraph and Telephone Corporation, Atsugi, Kanagawa, Japan

**Keywords:** human thermal perception, material recognition, tactual object perception, material perception, hand-object interactions, haptics

## Abstract

Some materials feel colder to the touch than others, and we can use this difference in perceived coldness for material recognition. This review focuses on the mechanisms underlying material recognition based on thermal cues. It provides an overview of the physical, perceptual, and cognitive processes involved in material recognition. It also describes engineering domains in which material recognition based on thermal cues have been applied. This includes haptic interfaces that seek to reproduce the sensations associated with contact in virtual environments and tactile sensors aim for automatic material recognition. The review concludes by considering the contributions of this line of research in both science and engineering.

## Introduction

A common daily experience is that some materials feel colder to the touch than others. Take metal for example. It generally feels colder than wood, even when both of them are at room temperature. The coldness of an object at room temperature is distinct from the object's physical temperature. The contribution of the perceived coldness to the recognition of materials was demonstrated by German psychologist David Katz in the 1920s.^1^

**Figure 1.**
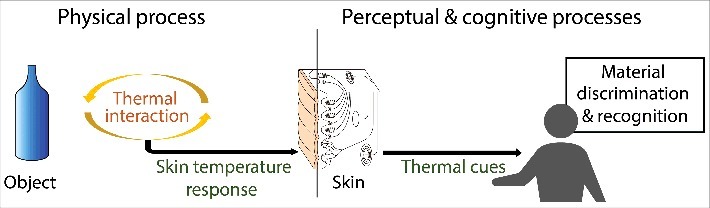
Physical, perceptual, and cognitive processes involved in material recognition based on thermal cues. In the physical process, the thermal interaction between the skin and the object elicits change in skin temperature, which is a function of the object's material composition. In the perceptual process, the change in skin temperature activates thermoreceptors and the coldness perceived is used as a cue for material recognition. In the cognitive process, the perceived coldness is classified into a certain material category to reach a material judgment.

Material recognition based on thermal cues involves physical, perceptual, and cognitive processes (see Fig. 1). The physical process refers to the thermal interaction between the skin and the object touched by the hand. Thermal cues that assist us in identifying an object arise from changes in skin temperature during hand-object interaction, which in turn depend on the object's material composition and other physical factors related to the skin, the object, and the skin-object interface. The perceptual process concerns our ability to perceive these changes in skin temperature and use them as cues for material discrimination and recognition. This ability is related to human thermal sensibility and depends on the physical characteristics of the object in contact. The cognitive process is related to the mapping between the perceived coldness and the internal representation of materials. This internal mapping determines how people classify the perceived coldness into a certain material category and reach a material judgment. Note that while the perception of coldness plays an important role in material recognition by touch, the perception of other material properties, such as surface texture, compliance, and friction, also affects people's material judgments.^2-5^

The importance of advancing our knowledge of how humans recognize materials on the basis of thermal cues is twofold. On the fundamental side, the more knowledge we have, the better we can understand tactual object perception, which is one of the most important and reliable sources of the information we use to understand the world around us. On the applied side, it will expand our knowledge base for the development of haptic interfaces that present information about the properties of objects encountered in real or simulated environments. By providing the thermal cues that are normally experienced by the hand during hand-object interactions, a more realistic image of the object can be created to enhance the user experience. At the same time, this line of research will also contribute to the development of tactile sensors that facilitate automatic object identification. By analyzing the heat transfer during the contact between a tactile sensor and the object to be identified, the object's material composition, which often cannot be directly inferred from its appearance, can be ascertained to facilitate object identification.

This review provides an overview of what we currently know about material recognition based on thermal cues. The primary focus is on the physical, perceptual, and cognitive aspects of material recognition based on thermal cues. Thermal displays that have been developed to facilitate object identification and discrimination will also be summarized, as well as tactile sensors for automatic object identification systems based on the heat transfer during contact.

## Physical process

The resting temperature of the skin is typically higher than the ambient temperature of objects encountered in the environment.^6^ When the hand is brought into contact with an object, the heat is conducted out of the skin and the corresponding changes in skin temperature are functions of the object's material composition and other physical factors related to the object, the skin, and the skin-object interface. The change in skin temperature during contact determines the perceived coldness of an object touched by the hand and is the key to material recognition based on thermal cues. To characterize the changes in skin temperature upon contact, empirical and theoretical models are used to predict the changes in skin temperature under various contact scenarios. This section starts with an overview of the physical factors involved in the heat transfer process, followed by a discussion of how these factors influence skin temperature responses during contact. Finally, thermal models for hand-object interactions are introduced.

### Physical factors that influence the heat transfer process

The thermal interaction between the skin and the object is dominated by heat conduction and is a transient process. For such a process, thermal conductivity and heat capacity are two important basic thermal properties to consider. Thermal conductivity determines how much and how fast the heat extracted from the finger spreads through the object in contact. A high thermal conductivity allows the object to extract more heat from the finger. Heat capacity determines the amount of extracted heat required to raise the temperature of the object in contact by one degree. A high heat capacity means that the heat from the finger does not warm up the object very much, which enables the object to continue extracting heat from the finger. Contact coefficient is directly related to the degree of coldness that would be perceived when touching an object.^7^ As a square root of the product of thermal conductivity and heat capacity, a material's contact coefficient represents its ability to conduct and store heat and in turn its capacity to extract heat from the finger during contact. It is the property that determines the contact temperature of two bodies brought into contact.^8^ The contact temperature is lowest for materials with high contact coefficients, which explains why metal feels cold (see Table 1 and Fig. 4A).

**Table 1.** Thermal properties of the skin and four common materials.

Material	Skin	Aluminum	Glass	Acrylic	Foam
Thermal conductivity *k* (W/mK)	0.37	237	1.38	0.20	0.19
Heat capacity *ρc* (J/m^3^K)	3.7*10^6^	1.87*10^6^	1.63*10^6^	1.72*10^6^	0.25*10^6^
Thermal diffusivity k/*ρc* (m^2^/s)	1*10^−7^	1267*10^−7^	8.47*10^−7^	1.16*10^−7^	7.6*10^−7^
Contact coefficient (*kρc*)^1/2^ (J/m^2^s^1/2^K)	1.17*10^3^	24*10^3^	1.50*10^3^	0.59*10^3^	0.22*10^3^

An object' geometry also has an influence on the heat transfer process during contact. Just consider, for example, the temperature difference one feels when touching a piece of aluminum foil and an aluminum block. The thickness of an object is important because the heat transfer within it during contact occurs mainly in the direction perpendicular to the contact surface.^9^ A thick object generally extracts more heat from the skin than a thin one made from the same material does, because of its better heat storage capacity due to its larger mass.

The skin factors involved in the heat transfer process during contact include its thermal properties, anatomical structure, and the effects of blood perfusion and metabolic heat generation.^10,11^ With its low thermal conductivity and low thermal diffusivity (see Table 1), skin is a good thermal insulator. Therefore, the changes in skin temperature during contact are localized to the contact area^12^ and there is a huge temperature gradient within the skin. The skin is composed of two principal layers: the epidermis and dermis, each of which has slightly different thermal properties.^13^ Within the dermis, there are thermoreceptors that respond to the changes in skin temperature during contact (see Section “Human thermal sensation” for details) and blood vessels that provide nourishment to the cells. Lying below the dermis is the hypodermis, which is the subcutaneous tissue that attaches the skin to underlying bone and muscle and supplies it with blood vessels and nerves. Because of these properties, the skin is often modeled as an inanimate material with a constant heat supply for heat transfer analysis (see Section “Thermal modeling” for details).

**Figure 2.**
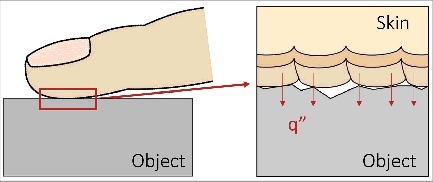
Heat transfer across skin-object interface during contact. When the skin and the object are brought into contact, only a small fraction of their surface areas is actually in contact because of the nonflatness and roughness of the contacting surfaces. This limited contact area restricts the amount of heat that can be transferred across the interface, and the degree of restriction is defined by thermal contact resistance.

The condition of the skin-object interface affects the heat transfer process during contact mainly through the thermal contact resistance presented at the skin-object interface. When two surfaces are brought into contact, only a small fraction of their surface areas is actually in contact because of the nonflatness and roughness of the contacting surfaces (see Fig. 2). This limited contact area restricts the amount of heat that can be transferred across the interface, and the degree of restriction is determined by thermal contact resistance. Thermal contact resistance depends on a number of variables, including the surface roughness, contact force, compliance, and the thermal conductivity of the skin and object in contact.^14^ This explains why in daily experience the sensation of cooling is more vivid if a finger presses an object surface harder during contact and why smooth surfaces generally feel colder to the touch than rough ones. Several methods have been proposed for estimating the thermal contact resistance for the contact condition involving the finger pad and a surface.^15-17^

Lastly, the initial temperatures of the skin and the object are important factors to consider for material recognition based on thermal cues. Their initial temperatures determine the direction and the amount of the heat exchanged during contact. When the resting temperature of the skin is lower than the temperature of objects encountered in the environment, the heat will be transferred from the object to the skin and warmness will be perceived upon contact instead of coldness. For both heat transfer directions, the amount of heat exchanged is positively related to the temperature difference between the skin and object. When there is no temperature difference between the two, there will be no change in skin temperature during contact and thus no thermal information for material recognition.

### Skin temperature responses during contact

**Figure 3.**
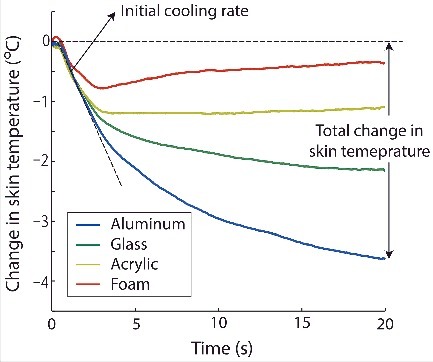
Changes in skin temperature when touching 4 common materials: Aluminum, glass, acrylic, and foam for 20 s. These cooling curves typically take a similar form, with a rapid temperature drop at the moment of contact and a slower change as time passes. Materials with high contact coefficients, such as metal, generally elicit a higher initial cooling rate and a larger total change in skin temperature than those with low ones. For materials with low contact coefficients, such as foam (red line), the skin temperature tends to start to increase again slowly after it has reached its lowest level, resulting in a V-shaped temporal profile.

During hand-object interactions, different materials will produce different cooling curves for the skin as shown in Fig. 3. These cooling curves typically take a similar form, with a rapid temperature drop at the moment of contact (initial phase) and a slower change as time passes (late phase). As can be seen in Fig. 3, the cooling curves of aluminum, glass, acrylic, and foam are distinct. The differences among them are often summarized with two curve features: the initial cooling rate and the total change in skin temperature throughout the contact period.^18^ Materials with high contact coefficients, such as metal, generally elicit a higher initial cooling rate and a larger total change in skin temperature than those with low ones. The difference in these cooling curves is the physical basis of the difference in perceived coldness among materials of the same physical temperature, and it also demonstrates that material recognition is possible with thermal cues.

The cooling curves associated with making contact with materials are often modeled as Newtonian cooling curves,^7,18-20^ which were originally used to model the cooling of a solid inanimate body to the temperature of the environment.^21^ A Newtonian cooling curve is a first-order exponential decay function. The inverse of the time constant derived with this model is related to the logarithm of the contact coefficient of the materials,^7^ which is consistent with the observation that a material with a higher contact coefficient causes faster skin cooling. Studies have also approximated the cooling with a modified Newtonian cooling curve, which is a second-order exponential decay function that can better describe the two-phase nature of the cooling.^22-24^ In this model, the first, shorter time constant describes the instantaneous skin temperature responses in the first few seconds of contact (initial phase), which is presumably related to the cooling of the very superficial epidermal layer and primarily thermocouple dynamics. The second, longer time constant describes the skin temperature responses in the late phase and is presumably related to effects resulting from prolonged contact, such as the cooling of the deeper dermal layers of the finger and the effects of metabolic heat generation and blood perfusion. For materials with a high contact coefficient, the cooling process is overall a lot quicker and dominated to a greater extent by the first, shorter time constant. On the other hand, for materials with a smaller contact coefficient, the cooling process is relatively slow and both the first and second time constants are longer, with the very long second time constant dominating the overall cooling process to a greater extent.

Another factor besides material composition that influences the skin temperature response during contact is object geometry. In general, a thick object tends to elicit a higher initial cooling rate and larger total change in skin temperature than a thin one made from the same material. Depending on the material composition and the thickness of the object, there are three possible responses in the late phase of contact. In general, for materials with high contact coefficients, such as metal, the skin temperature tends to continue decreasing at a lower rate or approach an asymptote as time increases. On the other hand, for objects that are very thin (e.g., 1-mm thick) or made from materials with low contact coefficients, such as foam, the skin temperature tends to start to increase again slowly after it has reached its lowest level, resulting in a V-shaped temporal profile as shown by the cooling curve of foam (red line in Fig. 3).^25^ The V-shaped temporal profile reflects the heating of the object during contact by the heat transferred to and in turn accumulated in it. For objects that are very thin, it is easy to observe the V-shaped temporal profile because the heat accumulation effect is reflected in the object's temperature within a short time. For materials that have a low contact coefficient (e.g., foam), the heat accumulation would be limited to the region near the contact surface; therefore, V-shaped temperature responses are easily observed, regardless of thickness.

Contact force also exerts its influence on the skin temperature responses.^26-28^ For contact force ranging from 0.1 to 6 N, the decrease in skin temperature is greatest between 0.25 and 0.35 N and from 4 to 6 N and smaller between 0.5 and 4 N.^27^ This nonlinear relationship indicates that the influence from the contact force does not merely arise through the thermal contact resistance, which suggests that the decrease in skin temperature is positively related to the contact force. The greatest decrease in the range of 0.25–0.35 N is presumably related to the collapse of blood vessels in the region of compression, which drives the blood away from the contact area to capillaries under the nail bed^24,29^ and causes a decrease in skin temperature that the thermal contact resistance model does not account for.

The effect of the object's surface roughness on the skin temperature responses is more complicated than that predicted by the thermal contact resistance model, which suggests an inverse relationship between surface roughness and the decrease in skin temperature; that is, high surface roughness would result in a smaller decrease in skin temperature. An investigation with copper surfaces whose roughness ranges from 165 to 216 µm has shown that there is a small but consistent decrease in skin temperature as a function of the surface roughness of the object in contact with the hand.^27^ This contradiction to the theoretical prediction points to the possibility that the finger may be able to deform more around a textured surface with higher roughness, which would result in a larger contact interface and thus a greater decrease in skin temperature.

### Thermal modeling

Thermal modeling is a way to systematically analyze the relationship between the physical factors involved in the heat transfer process to the skin temperature responses during contact. There are several ways to model the thermal interaction between the skin and an object during contact, starting with the assumption that the skin is an inanimate material to the inclusion of the effects of blood perfusion and metabolic heat generation, with the skin-object interface condition and the geometries of the finger and object precisely specified.

For short contact periods, the skin and the object can be modeled as semi-infinite bodies, which are idealized bodies with a single plane surface extending to infinity in all directions.^8,30,31^ Such an assumption is reasonable because within a short contact period the temperature disturbance within the object due to the thermal interaction is limited to the superficial layer, so the actual object geometry does not affect the heat transfer process. Generally speaking, a semi-infinite body assumption is valid under the condition that the Fourier number is less than 0.05:^8^

(1) $$Fo=\frac{\alpha t}{L_{c}^{2}}$$

where α is the thermal diffusivity of a solid body, *t* is the contact period, and *L*_c_ is characteristic length, which is the length through which conduction occurs. Based on (1), the validity of the semi-infinite body assumption can be estimated for any contact scenario. For the heat transfer process at the skin side, if we treat the skin as an inanimate material with a characteristic length (skin thickness) of 2.5 mm, the semi-infinite body assumption is valid for the skin for a contact period less than 3 s, which corresponds to the initial phase of the skin temperature response during contact (see Section “Skin temperature responses during contact” for details).

**Figure 4.**
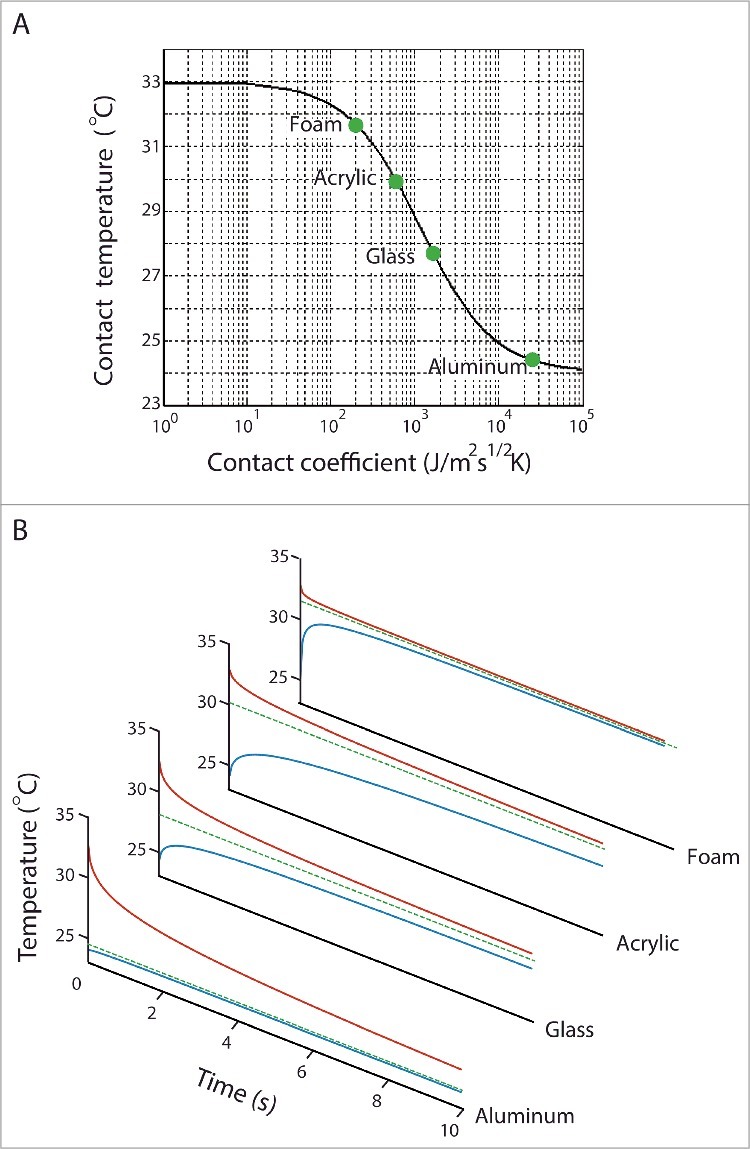
Temperature responses when touching 4 common materials predicted by (A) the semi-infinite body model and (B) the revised model. The semi-infinite boy model predicts the surface temperatures of the skin and object would change instantaneously to a contact temperature at the moment of contact and maintain constant throughout the contact duration (green dots and green dashed lines). The revised model takes into account the thermal contact resistance at the skin-object interface and provides a more realistic prediction on the skin (red line) and object surface (blue line) temperature responses.

Based on the semi-infinite body assumption, the heat transfer process during hand-object interactions can be simplified as “two semi-infinite bodies in contact”.^32-34^ This model concerns only the most basic factors of the heat transfer process during contact, namely the initial temperatures and the thermal properties of the skin and object. It predicts that the surface temperatures of the skin and object would change instantaneously to a contact temperature at the moment of contact and would maintain constant throughout the contact duration. Contact temperature *T_c_* can be calculated as:

(2) $$T_{c}=\frac{T_{object,i}{\left(k\rho c\right)}_{object}^{1/2}+T_{skin,i}{\left(k\rho c\right)}_{skin}^{1/2}}{{\left(k\rho c\right)}_{object}^{1/2}+{\left(k\rho c\right)}_{skin}^{1/2}}$$

where *(kρc)^1/2^* is the contact coefficient, *T_object,i_* is the initial temperature of the object, and *T_skin,i_* is the initial temperature of the skin. The thermal property that governs this process is the contact coefficient (see section “Physical factors that influence the heat transfer process” for details). It acts as a weighting factor that determines whether the contact temperature will more closely approach the initial temperature of the skin or object. Materials with high contact coefficients would result in a lower contact temperature as shown in Fig. 4A. Although the prediction offered by the “two semi-infinite bodies in contact” model differs from the empirical data, which show that at the moment of contact the surface temperatures of the skin and object change with time^35^ (Fig. 3), this model provides a first approximation of the heat transfer process during contact and is useful in predicting the perceived coldness of any given material.

The “two semi-infinite bodies in contact” model can be improved by taking into consideration the thermal contact resistance at the skin-object interface, which accounts for the influences from the contact force and the surface textures of the skin and object.^28,36^ Adding thermal contact resistance to the “two semi-infinite bodies in contact” model provides a more realistic prediction on the time course and amplitude of the skin temperature change during contact (Fig. 4B). The modified model performs well in predicating the initial cooling rate, but tends to overestimate the total changes in skin temperature throughout the contact period.^25^ Its performance reflects the limitation of the semi-infinite body assumption, which is only valid for short contact period. The overestimation of the temperature change is also related to the assumption that the skin is an inanimate material. In the actual heat transfer process, the skin temperature won't decrease as much because of the effects of blood perfusion and metabolic heat generation.

To model the skin temperature response at later phase of contact with the object, several studies have taken into consideration its geometry. The LC model proposed by Ho^25^ assumes the object in contact is a lumped system, that is, the temperature of the object is spatially uniform at any instant during the heat transfer process. This analytical model can precisely predict the skin temperature responses for thin objects or objects made from materials with high conductivities. The model proposed by Bergmann Tiest^37^ also concerns the influence from the object geometry. It is a one-dimensional finite element model which assumes a constant finger temperature to account for the effect of blood perfusion. It predicts that thick objects would extract more heat from the finger during contact and thus feel colder to the touch than thin ones. However, the model cannot predict the skin temperature responses during contact because of its constant finger temperature assumption.

To improve the accuracy of the thermal response prediction, studies have proposed models that consider the effect of metabolic heat generation and blood perfusion^38-41^ or divide the skin into several layers based on its anatomical structure.^9,41-43^ To date, various thermal models have been proposed for modeling the heat transfer process during hand-object interactions (for a full review, see ref. 17). The choice of assumptions depends on the particular contact scenario intended for analysis and its validation in experimental studies.

### Summary

The physical process of hand-object interactions is a fundamental of material recognition based on thermal cues. When an object is touched by the hand, the thermal, surface, and mechanical properties and the geometry of the object in contact are involved in the heat transfer process between the skin and object. These factors exert their influence on the change in skin temperature, which is the key to successful material recognition.

While the physical properties of common objects are easy to estimate, the changes in skin temperature under different scenarios are relatively difficult to obtain because of the time and effort required for the measurement. Because of this, the empirical skin temperature response data available so far have generally been obtained in experimental room settings using material samples with minimized variation in other factors, such as surface texture and size. These empirical data provide straightforward observations of how the skin temperature responses changes as a function of the physical factors manipulated in the experimental settings and are useful in identifying the relative importance of each physical factor. However, it can only characterize the skin temperature responses under the contact scenarios examined.

Thermal modeling can overcome the limitations of the empirical method as it is able to predict the thermal responses of the skin as it makes contact with an object under different contact scenarios. However, thermal models are based on different kinds of assumptions, each of which is only suitable for certain situations. Therefore, it is important to choose the one that fits the contact scenarios intended to be simulated. Nevertheless, the skin temperature responses during brief contact can be well-predicted by a model incorporating the semi-infinite body assumption with consideration of thermal contact resistance. This helps us to understand how people infer the material composition of objects with incidental contact, which is a situation that often happens in daily life – When people navigate through the surroundings, they often make contact with objects incidentally and the contact is only for a short period of time.

## Perceptual and cognitive processes

When the hand makes contact with an object, the change in skin temperature is encoded by thermoreceptors in the skin and transmitted to the central nervous system to assist in object recognition. As the resting temperature of the skin is typically higher than the ambient temperature of objects encountered in the environment, it is the cold thermoreceptors and afferent units that signal the decrease in skin temperature and the coldness perceived that characterizes the material touched by the hand. On the basis of the perceived coldness upon contact, people are able to discriminate and recognize materials touched by the hand. In material discrimination, the process involves direct comparison of the perceived coldness upon contact. In material recognition, it requires the mapping between the perceived coldness and the internal representation of the materials. This section provides an overview of human thermal perception. It covers how the human thermosensory system processes temperature information, followed by a review of the literature regarding people's performance on material discrimination and recognition based solely on thermal cues.

### Human thermal sensation

In the realm of human thermal sensation, warmness and coldness are mediated by separate channels.^44^ Warm sensations result from the activities of warm receptors, which are mainly innervated with slow conducting small unmyelinated C fibers. Cold sensations result from activities of cold receptors, which are mainly innervated with fast conducting small myelinated Aδ fibers.^45^ Because of the difference in nerve innervation, the conduction velocity of cold receptors is much higher (5–30 m/s) than that of warm receptors (0.5–2 m/s).^46^ The density of thermoreceptors varies from one body region to another, but cold receptors are always more numerous than warm receptors.^45^

Warm and cold receptors show both static and dynamic responses that represent the adapting temperature and the change in temperature, respectively. In the neutral thermal zone between 30 and 36°C, both types of thermoreceptors discharge spontaneously at low rates and no thermal sensation is noted. When the skin temperature drifts away from this neutral thermal zone, the relative discharge rate of the warm and cold receptors changes. In general, increases in skin temperature cause warm receptors to fire, and decreases in temperature result in cold receptors discharging. Warm receptors respond to a temperature range between 30–50°C with peak intensities around 45°C. Cold receptors respond to a temperature range between 5 and 43°C with peak intensities between 23–28°C.^45,47^ When the skin temperature rises above 45°C or falls below 15°C, nocioceptors respond to the extreme thermal stimuli, which results in pain.

Sudden changes in skin temperature, such as those elicited by touching an object surface with the hand, evoke dynamic responses in thermoreceptors. The warm receptors respond to sudden heating with a transient increase in the discharge frequency of action potentials. The peak responses tend to occur within 1 to 2 s after the end of the dynamic stimulus, and the peak frequency depends on the rate of temperature change, the magnitude of the thermal increment, and the adapting temperature of the skin.^48^ Cold receptors show similar response patterns, but their peak responses usually occur well before the ends of the temperature changes,^49^ indicating that they respond more readily to transient changes than warm receptors do. For both warm and cold receptors, the peak responses are followed by a rapid decline in frequency during the early stages of the new temperature level.^50^

The action potentials from the thermoreceptors are transmitted by the small-fiber spinothalamic system, and the neurons on which the spinothalamic fibers terminate have huge receptive fields.^51-53^ These thalamatic neurons further relay the thermoreceptive activity to the dorsal posterior insula, which has an antero-posteriorly organized topographic map of thermoreceptive activity directly correlated with warming or cooling in skin.^54,55^ The subjective evaluation of the thermal stimuli—the feeling elicited in each person by them—depends on post-processing of the information from the dorsal posterior insula in the right anterior insula and orbitofrontal cortex.^54^ In essence, the insula functions as the primary thermosensory cortex and is responsible for the discrimination and localization of temperature sensations in humans.

Humans are sensitive to changes in skin temperature, and the sensitivity is especially remarkable for cooling. On the thenar eminence at the base of the thumb, human subjects can resolve a difference of 0.02–0.07°C in the amplitudes of two cooling pulses or 0.03–0.09°C in those of two warming pulses.^56,57^ The absolute threshold for detecting a change in skin temperature, which would be the situation when the hand touches an object, varies at different body sites. The face, especially the lips, is the most sensitive region and the extremities are the least sensitive. Within the hand itself, there are local variations in thermal sensitivity. For example, the thenar eminence has superior warm and cold sensitivity compared to the fingertips. When the skin temperature is maintained at 33°C, the absolute warm and cold thresholds at the thenar eminence are 0.20 and 0.11°C, respectively, while those at the index finger are 0.55 and 0.30°C, respectively.^58^ In general, all body regions are more sensitive to cold than to warm stimuli, and the better a site is at detecting cold, the better it is at detecting warmth. This superior sensitivity for the detection of temperature changes indicates that humans are well-equipped for recognizing object material based on thermal cues.

When the skin temperature changes very slowly, with the rate being less than 0.1°C/s, an observer can be unaware of a change of up to 4 or 5°C, provided that the temperature remains within the neutral thermal zone of 30–36°C.^59^ The reduction in sensitivity that occurs with slower rates of temperature change is related to adaptation, that is, the loss of responsiveness to thermal stimulation as a result of continuous exposure to the stimulus. This is a frequently experienced phenomenon in daily life. For example, the sensation of warmth aroused when one steps into a bath gradually diminishes and may eventually disappear even though the water's temperature is kept constant. Adaptation is one of the most predominant characteristics of human temperature sensation. Due to adaptation, there is no fixed reference point for temperature perception. For this reason, humans are not very good at judging absolute temperature. In a study that investigated whether veridical perception of physical object temperature occurs in humans, participants were presented with a test stimulus to one hand and requested to find a matching temperature with the other hand after the two hands had adapted to different temperatures.^60^ A difference between the test and matching temperatures indicated a deviation from the veridical perception. Tritsch^60^ found that the veridical perception of object temperature is assured for objects whose temperature is much higher or lower than that of the skin. For objects whose temperature is close to that of the skin, the temperature sensation elicited deviates from veridical perception, indicating that both the object's temperature and change in skin temperature contribute to the temperature sensation elicited. The deviation from the veridical temperature perception in this temperature region makes recognition of object material based on thermal cues possible, as the material differences would be unrecognizable if we were only to sense the physical temperature of the materials.

Human temperature sensation has been shown to have good spatial summation and poor localization for thermal stimuli at low intensities.^61-66^ This is hardly noticed in daily experience because concurrent tactile inputs can facilitate thermal localization. For example, when the hand makes contact with an object, the change in skin temperature and the deformation of the skin activate both thermoreceptors and mechanoreceptors located in the skin. Localization of thermal cues during hand-object interactions has been studied using materials that span a wide range of thermal properties. Using this procedure, Ho and Jones^32^ determined the accuracy with which subjects could identify which of three fingers on one hand was in contact with a material that felt different (i.e., the target) from the material (i.e., distractor) that was in contact with the other two fingers. The ability to localize differences in the thermal responses of the fingers was poor (57% correct) and depended on both the thermal properties of the target and the distractor materials. In general, the performance was better when the difference in the contact coefficient was large between the target and the distractor.

The poor performance in localizing a material based on thermal cues is related to thermal referral, in which the interaction of thermal and tactile inputs leads to mislocalization of thermal sensations when adjacent parts of the skin are differentially stimulated.^67-70^ This phenomenon was first demonstrated by Green.^67^ When observers touched three stimulators simultaneously with the index, middle, and ring fingers of one hand, but only the outer two stimulators were cooled or heated, thermal uniformity was felt at all three fingers, with the intensity perceived as lower than the physical intensity applied to the outer two fingers.^70^ Thermal referral can be thought of as a phenomenon that reflects how thermal and tactile modalities coordinate to resolve incoherent spatial information acquired during hand-object interactions. It can facilitate object recognition, as it compensates for the discontinuity in the thermal properties of the sites in contact and creates a unified perceptual experience that is coherent across thermal and tactile modalities.

Besides the influence from the tactile inputs, temperature perception is also affected by visual information. For example, the prevailing “red-warm/ blue-cold” association has been shown to bias people's perception of environmental temperature – red or blue room lighting can make a person feel warmer or cooler.^71,72^ However, when it comes to touch, which is the case of the temperature perception elicited by touching an object, the effect of color is not as straight-forward. Ho, Iwai, Yoshikawa, Watanabe and Nishida^73^ demonstrated that a blue object would feel warmer to touch than a red object of the same physical temperature. This effect apparently opposes the common conception of red-hot/blue-cold association. The contradictory results have been interpreted as a contrast effect, that is, the prior expectation based on the red-hot/blue-cold association is integrated with direct temperature inputs in a way that emphasizes the “contrast” between the two, making the perception opposed to the expectation.^73-75^

### Material discrimination

Humans are able to discriminate materials based on thermal cues. In 1925, Katz^1^ demonstrated that people are able to order room temperature materials that span a wide range of thermal properties from “colder” to “warmer.” In this colder-warmer order, metals are typically at the cold end, while fabrics are at the warm end, and the order variation is extremely small across individuals. The discrimination between materials becomes more difficult if their temperature is very similar to the skin temperature, as in such situations the heat transfer between the skin and the object is limited and thermal information for a material becomes unavailable (see section “Physical factors that influence the heat transfer process” for details). When their temperature is raised above the skin temperature, the perceived order of “warmer” and “colder” is reversed, with metals at the warm end and fabrics at the cold end. A similar warmer-colder discrimination can also be performed by touching the top surface of the material samples differing only in thickness. At room temperature, thicker samples are perceived colder than thinner ones. When the ambient temperature is raised to 40°C, the thermal sensation is reversed, with thinner samples feeling colder than thicker ones.^76^

Without a doubt, certain differences in thermal properties are necessary for people to discriminate materials based on the perceived coldness upon contact. With a set of four disks made from copper, stainless steel, glass, and polyvinyl chloride (PVC), Dyck, Curtis, Bushek, and Offord^77^ found that the pairs of disks that normal healthy subjects can reliably distinguish on the palm of the hand as “cold” and “warm” were copper and PVC and copper and glass. Similar results are reported in refs. 35, 39, and 42. To further quantify the difference in thermal properties required for reliable discrimination, Jones & Berris^78,79^ examined the difference required for reliable discrimination in terms of thermal conductivity and heat capacity, which are two of the most important basic thermal properties involved in the heat transfer process during hand-object interactions (see section “Physical factors that influence the heat transfer process” for details). Their results suggested that a difference in thermal conductivity on the order of 70% or a difference of a factor of four in heat capacity is necessary for discrimination. Later, Ho and Jones^32^ estimated the difference required in terms of contact coefficient, which is the property directly related to the degree of coldness perceived when touching an object (see section “Physical process” for details). They found that people were able to reliably discriminate between materials when the difference in their contact coefficients differed by a factor of three or more. Similar results were obtained with simulated materials as well.^39^ However, these differences in thermal properties can't fully represent human capacity in discriminating materials based on thermal cues, because the materials used in these experiments did not have close and equal spacing of thermal properties. To obtain a precise estimation of the discrimination threshold, Bergmann Tiest and Kappers^18^ used a device to artificially extract heat from the finger to simulate materials with regularly spaced thermal diffusivities. They found that subjects were able to discriminate between simulated materials with thermal diffusivities differing by 43%.

The way the hands and fingers interact with materials influences one's performance in material discrimination. Yang, Kwon and Jones^38^ have shown that when the fingertip of a single finger is presented with two simulated materials, people are unable to discriminate between them even when the ratio of the differences in the contact coefficient is extremely large (urethane : copper, 850: 1). In another experiment, Yang, Kwon and Jones^38^ presented one simulated material to the middle three fingers of one hand and another simulated material to only the middle finger of the other hand and asked subjects to discriminate which middle finger felt colder. They found that the ability to discriminate between materials was influenced by the concurrent thermal stimulation of the index and ring fingers. The performance was enhanced when the “cooler” of two simulated materials was presented to three fingers, but the discrimination become more difficult if the “less cool” of the two materials was presented to three fingers. In the latter condition, participants were unable to discriminate reliably between the simulated materials—a task that could be accomplished quite easily when only one finger of each hand was stimulated.^39^ These cases of decline in performance are related to the spatial characteristics of thermal perception, that is, human thermal perception is rich in spatial summation and poor in thermal localization (see Section “Human thermal sensation” for details).

The human ability to discriminate materials has also been surveyed based on the time spent to respond to the thermal properties of a material. It has been shown that it takes about 900 ms to identify whether a copper sample, which has a high contact coefficient, is present in an array of two samples (copper and wood), and that the time increases with the number of distractor stimuli (i.e., wood) in the array.^80^ The time taken to respond to the thermal properties of materials is similar to that associated with identifying whether a cold object is presented among a number of warm objects.^81^ However, it is significantly longer than the time associated with encoding other material properties, such as hardness or roughness, which is 400–500 ms on average when two items (i.e., a target (hard surface) and a distractor (soft surface)) are presented in an array. This discrepancy in processing time is due to the fact that temperature changes are conducted more slowly by the smaller diameter Aδ and c fibers than other material properties, which are processed by larger diameter Aβ mechanoreceptor fibers.^81^

The human ability to discriminate between materials based on thermal cues is based on the ability to discriminate between the temporal profiles of the skin temperature responses (cooling curves) when touching the materials (see Fig. 3). In an experiment using a device to artificially generate cooling curves to simulate materials, subjects were requested to select from two stimuli the one that cooled faster.^18^ It was found that both initial cooling rate and the end temperature of the cooling curve contributed to the discrimination. In particular, the discrimination seemed to more strongly depend on the initial cooling rate, as the discrimination threshold was found to be double with a rate that was twice as slow but remained almost unchanged when the temperature difference between the beginning and end of the stimulus was halved. The results indicate that both thermal cues were used for discrimination but the cooling rate seemed to be the most important. This is consistent with the findings that our sense of cold is more sensitive to transient changes than to absolute temperature and that rapid changes in skin temperature are very salient stimulus features in dynamic thermal stimulation.^82^

### Material recognition

Absolute recognition of materials based on thermal cues involves mapping the temperature changes perceived upon contact to the internal representation of the materials, which presumably proceeds based on the remembered cues associated with making contact with materials in prior experience. Although the nature of those cues has not yet been clarified, they are presumably related to the features of the skin cooling curve produced upon contact, such as the initial cooling rate or end temperature.^18^ Katz^1^ referred to this internal representation of materials as a temperature gestalt and suggested that generic temperature gestalten should be available for common materials such as metal, wood, and fabric, so that the recognition of these material can be done readily based on thermal cues. In a series of experiments, he demonstrated that presenting materials in high temperature or in unusual size, e.g., aluminum foil, or asking people to touch the materials for an extremely short time or while wearing a glove, distorted the material temperature gestalt. Under these atypical situations, recognition suffered most for materials that usually feel cool (i.e. those with high contact coefficient, such as metals). For warm and neutral materials (i.e. those with low contact coefficient, such as wood or fabric), the recognition was possible but with considerable difficulty. On the basis of these observations, Katz postulated that thermal information might be indispensable for those materials that give a cool impression but would merely play a facilitative role for materials producing thermally neutral or warm impression.

In more recent studies, humans' performance in absolute recognition has been investigated in the context of the development of thermal displays, which are devices aiming to simulate materials based on thermal cues (see section “Applications” for details). In those studies, subjects were often presented with a set of material samples prior to the experiment. They were instructed to touch the samples and attend to the thermal cues associated with each material. The formal test would not start until they became familiar with the temperature transients perceived when they touched the materials presented to them.^33,35,43,83-85^ This type of task requires memory of all temperature changes perceived during the preparation phase to correctly identify one temperature transient presented in isolation; therefore, the performance in fact depends on the materials selected in the set. For example, Ino, Izumi, Takahashi and Ifukube^84^ examined people's performance with a set of material samples including aluminum, glass, rubber, polyacrylate (PVC), and wood and reported an overall correct rate of 61%. The best performance was achieved with aluminum and wood (both over 80%). On the other hand, in the experiment conducted by Ho and Jones,^35^ copper, stainless steel, granite, ABS, and foam were used, and the results indicated an overall performance of 55%, with foam being the most easily identified material (100%) and copper the least (23%). The poor performance for copper in Ho and Jones^35^ resulted from the fact that another metal, stainless steel, was in the set of the materials tested. Since the thermal cues provided by copper and stainless steel were similar, that is, in Katz's words, both of them share the same generic temperature gestalten, subjects were not able to distinguish them. In another experiment performed by Caldwell and Gosney,^85^ subjects were asked to identify materials based on temperature transients that had been recorded from a teleoperated robotic hand as it made contact with a variety of objects (a cube of ice, a soldering iron, insulating foam, and a block of aluminum). The overall correct rate of this particular task reached 80%. This excellent performance presumably resulted from the large temperature transients they used to simulate materials, which were not maintained at the same room temperature (e.g., a cube of ice and a soldering iron).

Absolute recognition of material by touch in fact involves other tactual cues, such as surface texture and compliance. As such, it is expected that subjects' performance would be better with real materials than with simulated ones, as the former contains both thermal and tactual cues and the latter can only provide thermal cues. However, ample studies have shown that subjects' performance actually does not significantly differ between real and simulated materials.^35,43,84^ These studies indicate that thermal cues alone can achieve a recognition rate comparable to those encountered normally for identifying the material composition of objects.

In the experiments discussed so far, thermal cues were usually presented to the fingerpad of a single finger, which is quite different from the normal situation where multiple fingers or the whole hand are involved in the hand-object interaction. Given the pervasiveness of spatial summation in the thermal senses as discussed in section “Human thermal sensation”, the performance in fact improves as the number of fingers involved (contact area) increases. Yang, Kwon and Jones^38^ presented thermal cues to one, three, or five fingers. They found that when the thermal cues were presented to a single finger, the subjects achieved a recognition rate of 52% correct, which is comparable to that achieved in other studies. Performance improved to 63% and 67% correct with three and five fingers, respectively. Their results indicate that increasing the thermal field of view does facilitate performance in material recognition but that there is little further benefit associated with presenting cues to five as compared to three fingers.

### Summary

Humans are well-equipped for recognizing an object's material based on thermal cues. In particular, the ample amount of cold receptors in the skin, their superior sensitivity for detecting decreases in skin temperature, and their readily responses for dynamic changes allow humans to extract material information from objects readily. Adaptation, the predominant feature, seems to prevent humans from functioning as an accurate thermometer. However, its influence on object temperature perception in fact allows estimation of both absolute temperature and a change in skin temperature in different temperature ranges. In this way, it enables us to respond to extremely high or low temperatures that would cause skin damage and to detect changes from normal skin temperatures to facilitate material recognition.

Our capacity to discriminate materials based on thermal cues is mainly estimated in terms of the physical properties of objects, such as their contact coefficient or thermal diffusivity, which is straight-forward and useful for practical design purposes. On the other hand, the capacity has also been estimated in terms of the features of skin cooling curves, e.g., the initial cooling rate. This type of estimation relates the skin temperature response upon contact, which is directly input to the thermoreceptors, to human performance in material discrimination; thus, it can provide a hint about the thermal cues used for material discrimination.

Absolute recognition of materials based on thermal cues involves mapping the temperature changes perceived upon contact to the internal representation of the materials (temperature gestalt). When the hand-object interaction scenario differs from the normal conditions, such as when the object temperature is higher than the skin temperature or the object in contact is extremely thin, the performance degrades because the skin temperature responses deviate from the temperature gestalt held in the memory. This suggests that unlike color perception, which remains relatively constant under varying illumination conditions (color consistency, see ref. 86), there might not be any “material consistency” in material recognition based on thermal cues. Nevertheless, under normal conditions, humans exhibit fair performance in material recognition based on thermal cues. The performance is comparable between real materials, which contain all aspects of tactual cues, and simulated materials, which only contain the thermal characteristics of the real materials, indicating that thermal information alone is effective for material recognition.

## Applications

**Figure 5.**
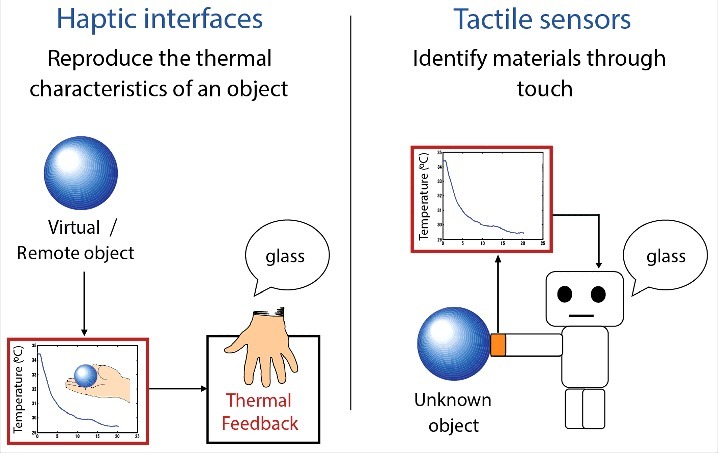
Two major applications for material recognition based on thermal cues. In haptic interface applications, the thermal characteristics of a virtual or a remote object can be reproduced by presenting thermal feedback generated based on the pre-recorded or predicted changes in skin temperature associated with touching the object. In tactile sensor applications, the material composition of an unknown object can be ascertained by analyzing the temperature changes of the sensory body, which are elicited by the heat transfer during the contact between the tactile sensor and the object.

Haptic interfaces—devices that communicate with the user's sensory system through tactile and force feedback—represent one of the major application domains for material recognition based on thermal cues. By reproducing changes in skin temperature upon contact in a thermal display, the thermal characteristics of an object and thermal sensations associated with the contact can be reproduced to assist users in recognizing a virtual object in virtual environments or a remote object handled by teleoperated robotic systems (Fig. 5).

Over the past 20 years, various thermal displays have been built to assist in material recognition by simulating thermal cues associated with contact (for a review, see ref. 17). These thermal displays typically consist of thermal stimulators, thermal sensors, and a temperature control system that is used to monitor and control the surface temperature of the displays. In the early stages of thermal display development, the characterization of the changes in skin temperature during contact was based on empirical data recorded during hand-object interactions. Although these empirical methods were straightforward, they were time consuming and labor intensive because numerous measurements had to be made in order to simulate every object and every environmental condition that could appear in a virtual scenario. This limitation was overcome when thermal modeling was introduced to predict the skin temperature changes during contact (model-based simulations, see Section “Thermal modeling” for more details). However, as thermal models typically involve assumptions and thus simplification of real-world situations, the rule of thumb for model selection is to choose one where the assumptions match the particular contact scenario intended to be simulated.

The performance of the thermal displays is commonly evaluated with physiological and psychophysical approaches. The physiological approach evaluates the ability of the display to elicit changes in skin temperature that are similar to those measured during contact with a real object. There is generally a good agreement between the changes in skin temperature with the real and simulated materials; however, these changes are often smaller than those predicted by the thermal models. This discrepancy presumably results from the localized nature of the change in skin temperature and the extra thermal contact resistance introduced by the skin-sensor-display interfaces. The psychophysical approach evaluates the accuracy of a thermal display in terms of its ability to generate appropriate temperature impressions of materials. This is usually evaluated by comparing the performance of users as they try to discriminate and recognize real materials and materials simulated by the thermal display. User performance is generally comparable for real and simulated materials in both material discrimination and recognition tasks. The overall correct rate for material recognition tasks is typically in the range of 50–60% for a set of five or six materials spanning a wide range of thermal properties (see section “Material recognition” for more details).

As the technology for material simulation matures, recent thermal display development has started to focus on further enhancing system performance by utilizing the properties of human thermal perception. For example, based on the well-known thermal referral illusion (see section “Human thermal sensation” for details), Sato^87^ proposed a technique that can create illusory thermal sensations at the fingerpad through thermal stimulation at the finger side. This technique allows the fingerpad to be free from direct contact with the thermal stimulator. Such a display can be easily integrated with other haptic devices, such as vibrators and electrotactile displays, to provide a holistic image of objects. Another example is a thermal display that utilized two characteristics of human thermal perception: low spatial resolution and dependence of detection threshold on adapting temperature.^88^ This display consisted of a 2 × 2 array of Peltier devices, of which cooling and warming are presented by different sets of 2 Peltier devices arranged diagonally. This configuration avoided the time required for a single Peltier device to switch between warming and cooling and because of the low spatial resolution of thermal perception, users felt that the entire surface is warm (or cold) even when only two Peltier devices were activated. In addition, prior to the presentation of warming (or cooling) stimuli, the corresponding Peltier devices slightly raised (or lowered) the adapting temperature of the skin to reduce the detection threshold for the upcoming warming (or cooling) stimuli. As a result, it took less time for users to detect the stimuli. As the limitations of the Peltier-based displays mainly came from the slow response of Peltier devices, these strategies provided a solution for providing rapid temperature changes required in thermal display applications.

Tactile sensors—devices that measure a given property of an object or contact event through physical contact between the sensor and the object^89^—represent another application domain for material recognition based on thermal cues. When a tactile sensor is brought into contact with an object, the object's material composition can be inferred from the temperature changes of the tactile sensor itself, just as how humans can recognize materials thermally during hand-object interactions (see Fig. 5).

In the early stages of thermal sensing technology, researches focused on the hardware development.^90-92^ The most basic configuration of a tactile sensor for thermal recognition consists of a heat source and a thermal sensor embedded immediately underneath its contact surface. The sensor body is usually heated prior to contact to create a temperature difference to the target object. This temperature difference induces thermal interaction between the sensor and the object and in turn elicits temperature changes at the sensor body, which are then used for material recognition. To enhance the system performance, the tactile sensors were often arranged in an array to ensure sufficient thermal contact^90,92^ and the choice and arrangement of heat sources and thermal sensors were optimized to reduce the response time of the tactile sensors.^91^

As technology for sensor hardware becomes mature, recent studies have started to incorporate thermal sensing to multimodal tactile sensing hardware to enhance recognition performance.^93-95^ At the same time, the development of tactile sensors for thermal recognition has switched from hardware-centric to algorithm-centric to improve the material classification performance. Classifying materials based on the sensor responses typically involves reference to a material database. Different types of information has been used for material classification: values that can be readily compared with the measurement data, such as the temperature transients^92^ and output responses of the tactile sensor's circuit;^91^ features that require pre-processing of the raw data, such as the time derivative or the time constant of the temperature transients;^90,96-99^ the thermal properties that need to be estimated from a thermal model.^100^ With the rise of matching learning, the data-driven approach has nowadays become the major algorithm for material classification. Material information gathered based on thermal sensing and/or multimodal sensing have been collected and trained with algorithms such as hidden Markov models (HMMs), support vector machines (SVMs), artificial neural networks (ANNS) etc.^98-100^

The accuracy of the thermal recognition ranges between 50 and 100%. The performance is influenced by various factors. A major factor is the duration of contact. Prolong contact contains temperature information in both early and late phases and thus yields better performance (See section “Skin temperature responses during contact” for details). Other factors, such as the initial temperature of the tactile sensor and the information used for classification (e.g., raw data or the time derivative of the temperature transients) also have an effect on the performance.^100^ For applications that utilize multi-modal recognition, i.e., recognition based on thermal and tactile information, generally better performance can be obtained.

In summary, thermal cues play an important role in the development of haptic interfaces and tactile sensor applications. In the development of haptic interfaces, thermal modeling is a common approach for characterizing the skin temperature changes during contact. By presenting the predicted temperature changes with a display, haptic interfaces can assist in creating a more realistic image of an object to enhance the user experience in virtual environments. On the other hand, in the development of tactile sensors, classification is commonly based on trained measurement data rather than thermal models. These tactile sensors could assist in automatic object identification by providing information about a target object's material composition, which cannot be readily inferred from state-of-the-art computer vision.

## Conclusion

The ability to sense temperature is vital to our life. It signals environmental conditions and helps to regulate the physiological conditions of our body. Besides its important role in homeostasis, our temperature sense has functional significance in environmental exploration and object recognition. The coldness or warmness felt by directly touching an object can provide information about the object's material composition and even geometry, which is something that can't be directly inferred from the visual information that we rely on most of the time. This unique characteristic makes material recognition based on thermal cues an important topic of study in both science and engineering fields.

The changes in skin temperature upon touching an object is the key to successful material recognition based on thermal cues. Accordingly, a great effort has been made to characterize the changes in skin temperature during contact through both direct measurement and thermal modeling. The analysis of cooling curves indicates the two-phase nature of the skin temperature responses. In the early phase, the skin basically can be treated as an inanimate object with semi-infinite dimensions, and its responses are well predicted by thermal models. For the later phase, the process becomes more complicated because of the involvement of blood perfusion, metabolic heat generation, and the object's geometry, and the prediction for prolonged contact still requires improvement. This two-phase nature of the skin cooling curve suggests that brief, incidental contact and prolonged, intentional contact should be considered independently in the processes involved in material recognition based on thermal cues.

How people perceive changes in skin temperature upon contact depends on the properties of human thermal perception. Because of the predominant adaptation effect, humans do not function as thermometers but have superior sensitivity for the detection of temperature changes. These properties have made humans well-equipped for recognizing object material based on thermal cues. The influences from the thermo-tactile and thermo-visual interactions on object temperature perception reflect the fact that thermal information is not processed independently for object recognition. As object recognition also involves perception of other sensory properties, it is important to understand how the brain integrates thermal and other sensory information to reach a material judgment.

People's performance in material recognition based on thermal cues is mainly evaluated in material discrimination and material recognition tasks. Material discrimination involves direct comparison of the perceived coldness upon contact. The minimum difference required for reliable discrimination has been estimated in terms of thermal properties. Generally speaking, a large difference in thermal properties, such as comparison between materials in different material categories, is required for reliable discrimination. Absolute material recognition based on thermal cues involves mapping between the perceived temperature changes to the internal representation of the material and thus heavily depends on prior experience and the set of materials presented. In general, the overall recognition rate depends on how distinct the thermal properties of the materials in the set are. For performance with individual materials, a material with an extremely low or high contact coefficients is easier to recognize than one with intermediate contact coefficients. People's performance in material discrimination and recognition has been tested with both real and simulated materials. The results show that simulated cues can be used as effectively as those associated with real materials. This presumably reflects the relatively simple processing capacities of the thermal senses. Thermal stimulation is encoded as being warm or cool, and it is then quantified in terms of intensity and duration. Therefore, as long as the thermal feedback provided by a thermal display reasonably characterizes the thermal interaction between the hand and an object, it should be as effective as real materials.

Material recognition based on thermal cues has two major applications. One is to facilitate material recognition in virtual environments with haptic interfaces, and the other is to assist automatic object recognition with tactile sensors. Both applications are based on the temperature transients elicited during contact. Haptic interfaces present the temperature transients associated with making contact to simulate the thermal characteristics of an object. As they serve to reproduce sensations associated with contact to human users, besides simply reproducing the temperature transients, researchers in this field also utilize the properties of human thermal perception to enhance their performance. On the other hand, tactile sensors measure the temperature transient upon touching a material and compare it to a database for classification. As their goal is to classify unknown materials effectively, efforts have been made to determine the best features, contact duration, and training algorithms to improve the recognition performance.

In summary, there has been considerable progress in understanding and modeling the heat transfer process during hand-object interactions. How much thermal cues can contribute to material discrimination and recognition has also been examined by various research groups. This research has contributed to a better understanding of the object temperature perception, and it also serves as a basis for engineering applications, such as haptic interfaces and tactile sensors.
